# Supplementary material for: Caregiver-Reported Economic Impacts of Pediatric Rare Diseases—A Scoping Review
Source: Healthcare (Basel). 2024 Dec 21;12(24):2578. doi: 10.3390/healthcare12242578 (PMC11727781; doi:10.3390/healthcare12242578)
Supplement: Supplementary file 1 [file healthcare-12-02578-s001.zip › Supplementary Table S1. Database Search Strategies.docx]

**Table S1. Sample Search Strategy**

| **Database** | **Search Strategy** | **Modifiers** | **Total Articles Returned** |
| --- | --- | --- | --- |
| ABI Inform | ((famil*) OR (parent*) OR (caregiver*) OR (guardian*)) AND ((cost of illness) OR (economic evaluation*) OR (economic burden) OR (burden of illness) OR (economic cost*) OR (economic impact*) OR (financial hardship)) AND ((paediatric) OR (child*) OR (adolescen*) OR (sibling*) OR (newborn*) OR (infan*)) AND ((rare disease*) OR (rare condition*) OR (orphan disease*) OR (orphan condition*) OR (ultra-rare disease*) OR (ultra-rare condition*) OR (ultra-orphan disease*) OR (ultra-orphan condition*)) | Abstracts; English language; peer-reviewed; published between 1 January 1983 – 04 April 2023. | 21,067 |
| CINAHL | ((famil*) OR (parent*) OR (caregiver*) OR (guardian*)) AND ((cost of illness) OR (economic evaluation*) OR (economic burden) OR (burden of illness) OR (economic cost*) OR (economic impact*) OR (financial hardship)) AND ((paediatric) OR (child*) OR (adolescen*) OR (sibling*) OR (newborn*) OR (infan*)) AND ((rare disease*) OR (rare condition*) OR (orphan disease*) OR (orphan condition*) OR (ultra-rare disease*) OR (ultra-rare condition*) OR (ultra-orphan disease*) OR (ultra-orphan condition*)) | Abstracts; English Language; Peer-Reviewed; Published between January 1983 – April 2023. | 23 |
| EconLit | ((famil*) OR (parent*) OR (caregiver*) OR (guardian*)) AND ((cost of illness) OR (economic evaluation*) OR (economic burden) OR (burden of illness) OR (economic cost*) OR (economic impact*) OR (financial hardship)) AND ((paediatric) OR (child*) OR (adolescen*) OR (sibling*) OR (newborn*) OR (infan*)) AND ((rare disease*) OR (rare condition*) OR (orphan disease*) OR (orphan condition*) OR (ultra-rare disease*) OR (ultra-rare condition*) OR (ultra-orphan disease*) OR (ultra-orphan condition*)) | Abstracts; English Language; Peer-Reviewed; Published between January 1983 – April 2023. | 2 |
| Medline | ((famil*) OR (parent*) OR (caregiver*) OR (guardian*)) AND ((cost of illness) OR (economic evaluation*) OR (economic burden) OR (burden of illness) OR (economic cost*) OR (economic impact*) OR (financial hardship)) AND ((paediatric) OR (child*) OR (adolescen*) OR (sibling*) OR (newborn*) OR (infan*)) AND ((rare disease*) OR (rare condition*) OR (orphan disease*) OR (orphan condition*) OR (ultra-rare disease*) OR (ultra-rare condition*) OR (ultra-orphan disease*) OR (ultra-orphan condition*)) | Abstracts; English Language; Publication Year 1983 – 2023. | 53 |
| PubMed | ((famil*) OR (parent*) OR (caregiver*) OR (guardian*)) AND ((cost of illness) OR (economic evaluation*) OR (economic burden) OR (burden of illness) OR (economic cost*) OR (economic impact*) OR (financial hardship)) AND ((paediatric) OR (child*) OR (adolescen*) OR (sibling*) OR (newborn*) OR (infan*)) AND ((rare disease*) OR (rare condition*) OR (orphan disease*) OR (orphan condition*) OR (ultra-rare disease*) OR (ultra-rare condition*) OR (ultra-orphan disease*) OR (ultra-orphan condition*)) | Publication Date 1983/01/01 – 2023/04/04; English | 477 |
| SCOPUS | ((famil*) OR (parent*) OR (caregiver*) OR (guardian*)) AND ((cost of illness) OR (economic evaluation*) OR (economic burden) OR (burden of illness) OR (economic cost*) OR (economic impact*) OR (financial hardship)) AND ((paediatric) OR (child*) OR (adolescen*) OR (sibling*) OR (newborn*) OR (infan*)) AND ((rare disease*) OR (rare condition*) OR (orphan disease*) OR (orphan condition*) OR (ultra-rare disease*) OR (ultra-rare condition*) OR (ultra-orphan disease*) OR (ultra-orphan condition*)) | Article title, Abstract, Keywords; Publsihed from 1983 – 2023; Added to Scopus Anytime; English language | 368 |
